# Exploring relationship pathways to prevent intimate partner violence among young women in Malawi

**DOI:** 10.1080/13691058.2025.2609888

**Published:** 2026-01-05

**Authors:** Audrey Pereira, Joseph Chunga, Juba Kafumba, Maxton Tsoka, Clare Barrington

**Affiliations:** a ISDC - International Security and Development Center, Berlin, Germany; b Department of Public Policy, University of North Carolina, Chapel Hill, North Carolina, USA; c Carolina Population Center, University of North Carolina, Chapel Hill, North Carolina, USA; d Centre for Social Research, University of Malawi, Zomba, Malawi; e Department of Health Behavior, Gillings School of Global Public Health, University of North Carolina, Chapel Hill, North Carolina, USA

**Keywords:** Intimate partner violence, cash transfers, poverty, gender norms, adolescence

## Abstract

International estimates of intimate partner violence (IPV) among adolescents and young women are high, indicating the need to address IPV prevention early in life. Structural economic interventions, such as household cash transfer programmes, have the potential to improve the wellbeing of youth who are not the direct recipients of the transfers themselves. However, few studies have addressed this topic in terms of youth romantic and/or sexual relationships. We conducted 39 in-depth interviews with young women aged 19–29 years in households participating in the Government of Malawi’s Social Cash Transfer Programme (SCTP) to examine relationship formation, IPV triggers and experiences, and help-seeking behaviours. We found that young women did not directly attribute SCTP effects to their intimate relationships or IPV experiences. Threats to masculinity and transgressions of women’s gender norms were key triggers of IPV, but specific triggers were linked to specific types of IPV. Furthermore, women sought help for non-IPV concerns more than IPV-related issues. Our results reveal there is a need to strengthen cash transfer programmes and layer them with tailored interventions for adolescents and young women in participant households to improve relationships and prevent IPV early in life.

## Introduction

Internationally, lifetime experiences of physical and/or sexual intimate partner violence (IPV) are high, ranging from 24 to 28% for young women aged 15–19 years, and 29% for those aged 20–24 years (Sardinha et al. 2022). In central and eastern sub-Saharan Africa, nearly one-third of ever-partnered girls report having experienced IPV in the past year (Sardinha et al. 2024). Within households, poverty, gender norms, and their interactions foster power imbalances and shape young women’s behaviours, intimate (romantic and/or sexual) relationships (henceforth, ‘relationships’) and IPV experiences (Buller et al. 2018; Gibbs et al. 2020; Heise 1998; Jewkes 2002; Moreau et al. 2019). Socio-cultural factors, such as stigma, shame and violence, and structural factors, including poverty and weak health and legal systems, prevent girls from challenging these norms (Maina et al. 2021). For instance, household economic hardship may lead young women to seek material support from male partners or engage in exploitive transactional sex. Resource theory predicts that women with fewer resources have lower bargaining power in relationships, impacting their ability to mitigate IPV or leave abusive relationships (Conroy 2014; Foa and Foa 1980; Meyer et al. 2024).

Structural economic interventions, such as cash transfer programmes, can be effective in improving adolescent wellbeing (Angeles et al. 2019; Handa et al. 2014; Lambon-Quayefio et al. 2024). In South Africa, cash transfers given to young women aged 13 to 20 years and their caregivers decreased physical IPV (Pettifor et al. 2016). These effects were mediated by delaying sexual debut and reducing the number of sexual partners, but these effects did not persist in the long-term (Groves et al. 2024; Kilburn et al. 2018). This design feature of directly targeting adolescents is different from large-scale, government-led programmes that target poor households, making it important to understand whether state-sponsored basic income support can achieve the same results as smaller more focused programmes. The Government of Zimbabwe’s Harmonised Social Cash Transfer programme targeting households with cash and protection services reduced any physical violence experience among youth (Chakrabarti et al. 2020). In the Government of Tanzania’s Productive Social Safety Net household cash transfer programme, the *Ujana Salama* component included livelihoods and skills training and grants conditional on attending training events. This additional component compared to cash alone reduced lifetime sexual violence risk among female youth in the intervention arm at endline and post intervention (Palermo et al. 2021; Waidler et al. 2025). However, these three studies measured experiences of any form of violence, rather than that perpetrated only by partners. More broadly, there is a need to understand in particular how cash transfers that target households affect relationships and experiences of IPV among young people in these households. There is also insufficient knowledge on girls’ and young women’s relationship processes including the circumstances in which young women enter relationships, relationship norms, IPV triggers and experiences, and consequent help-seeking behaviours.

To fill these gaps, we assessed whether and how the household-level Malawi Social Cash Transfer Programme (SCTP) influenced relationships and IPV among young women in these households. We also examined relationship formation, IPV triggers and experiences, and help-seeking behaviours through a relationship mapping process.

## Methods

### The Malawi social cash transfer programme (SCTP)

The Government of Malawi’s SCTP began in 2006. It targets ultra-poor, labour-constrained households, currently reaches over 1.3 million individuals, and is the largest social protection programme in the country (Malawi SCTP Evaluation Team 2014). Unconditional transfers are paid bi-monthly in cash to the heads of household. The amount of the transfer varies by household size but averages approximately MWK 8,500 (USD 4.50) per household per month (Malawi Government 2024). Economic vulnerability among SCTP households is extremely high – the total annual median per capita consumption averaged MWK 155,866 (USD 0.52) per day in 2022 (Malawi Government 2024).

An impact evaluation of the SCTP using a cluster randomised trial design was conducted between 2013 and 2015, when households received the SCTP earlier in 2013 (treatment) versus later in 2016 (control). Within each household, in addition to interviewing the adult SCTP participant, up to three young people aged 13–19 years in 2013 were administered modules on romantic partners, sexual relationships, health, and schooling, among others (Malawi SCTP Evaluation Team 2016). In June 2022, these young people (aged 19 to 29 years) were re-interviewed as part of a long-term follow up survey. Building on the quantitative survey, the current study was conducted with women to further explore cash transfers and relationship processes.

### Data collection

Data collection was conducted in Salima and Mangochi districts in July 2022. We randomly selected treatment and control clusters from the larger programme evaluation and interviewed young women in these clusters, ensuring equal numbers by district, treatment status, and age group (younger 19–24 years vs. older 25–29 years) (Table 1).

The first four authors trained six Malawian women interviewers on study aims, ethical considerations, and interview guides. All interviewers had been previously involved with data collection activities for the SCTP and were fluent in Chichewa.

The interview guide covered community and household context; norms about young people’s relationships; relationship dynamics, including relationship quality and stressors, IPV, and help seeking behaviours. We defined IPV as emotional, psychological, physical or sexual harmful behaviours and controlling behaviours in marriage, cohabitation, or any other form of union (Sardinha et al. 2022). Participants were also asked about experiences with, and perceptions of, SCTP on themselves and their households. All participants, regardless of reported experience of IPV to protect confidentiality, were provided with information on a variety referral services, including violence protection. Interviewers took notes during and audio-recorded interviews, which they later transcribed and translated to English.

### Data analysis

During data collection, the first author debriefed with interviewers to identify recurring themes. After data collection, the first author read and conducted quality control, requesting clarification on transcripts as needed. The first author then created visual trajectories of women’s relationships, especially noting economic vulnerability in entering relationships, relationship tensions, violence triggers, IPV experiences, help-seeking behaviours and leaving relationships (see examples contained in supplemental online Appendix A). Common stressors and triggers of violence identified included: infidelity, lack of male provision, women’s household responsibilities, and women denying sex.

We then developed a codebook based on broad themes in interview guide along with inputs from the visual trajectories (Gibbs 2018). Coding was conducted using ATLAS.ti (ATLAS.ti Scientific Software Development GmbH 2024). Codes were iteratively added and merged, and transcripts were re-coded as needed to ensure consistency after modifications to the codebook. Following coding, the first author developed code summary memos and a matrix of relationship pathways to primarily identify common patterns and themes across the interviews, and recontextualise the results to facilitate data reduction and interpretation. Differences in results by cash transfer treatment status based on the broader study evaluation were also assessed.

### Ethical approval

The University of North Carolina at Chapel Hill Internal Review Board and Malawi’s National Commission for Science and Technology’s National Committee for Research in Social Sciences and Humanities provided ethical approvals for this study (References: IRB # 19–1611 and # 20/04/12538, respectively). Informed consent was obtained from all participants, who had the right to withdraw from the study at any time. Each participant received a bag of sugar worth approximately USD 2 in appreciation of their participation. Transcripts were stored on a secure, password-protected server, accessible only to the researchers and deidentified before analysis.

## Results

### Participant characteristics

Descriptive characteristics of the sample are provided in Table 2. Women’s average age was 24.4 years and ranged from 19 to 29 years. Reported current or most recent partner’s age was approximately 30.3 years and ranged from 19 to 42 years. Most women had at least a primary education while only 23% had some secondary education. Twenty-seven women (69%) were currently married or in a relationship. Thirty-one (79%) women had children, and among those that had children, each had 1.9 living children on average. Polygamy was also practised in this setting.

### The Malawi SCTP and intimate relationships

We initially aimed to assess how the SCTP affected young women’s relationships and experiences of IPV, but these effects proved not to be salient themes in our interviews. None of the participants spoke of the effects of the cash transfer either on their daily lives or on their relationships. When probed, young women responded that their caregivers used transfer money mostly for household basic needs and that they had benefitted from these effects when they lived with their caregivers. However, few women stated that they still received help after leaving their caregivers households. One woman whose mother had provided her with some cash from the transfer explained,

I use it for my home such as buying food, soap, buying school materials (books, uniform, school fee, shoe, clothes) for my children and I keep the rest for emergencies such as buying drugs when I or the children get sick’ (R6, 23 years old)

Others who received help similarly used the money for basic needs and emergencies. Most women did not link the cash transfer benefits to their relationships even when asked. There were also no clear differences in our findings between young women who grew up in treatment (early cash transfer receipt) versus control (late cash transfer receipt) households. Therefore, in the rest of this paper, we present themes across all young women regardless of household’s treatment status.

### The context of relationship formation

We found that the overarching environment of living in extreme poverty affected decisions to enter relationships, relationship quality and IPV. When asked to narrate the story about how she met her husband, one woman shared that she entered her relationship because of ‘poverty … I had no beddings and it was hard to find food’ (R16, 23 years). Several other women also discussed how they accepted men’s proposals for sexual relationships or marriage because they lacked resources such as food and soap, describing their ‘hardships’ and ‘suffering’.

Finding a husband was also a means to have support without having to resort to sex work, as one woman stated, ‘I just wanted someone to be giving me financial support. I was in poverty so unlike going to bars, I preferred to get married’ (R15, 29 years), demonstrating women’s limited choices in this setting. However, not all women entered relationships solely out of economic need. While some women reported entering relationships for a combination of love and need, others reported love as the sole reason for their relationships. Nevertheless, poverty was an underlying theme in the interviews, affecting relationship dynamics and IPV experiences, as revealed in the stories below.

### Mapping pathways related to IPV

**Figure 1.**
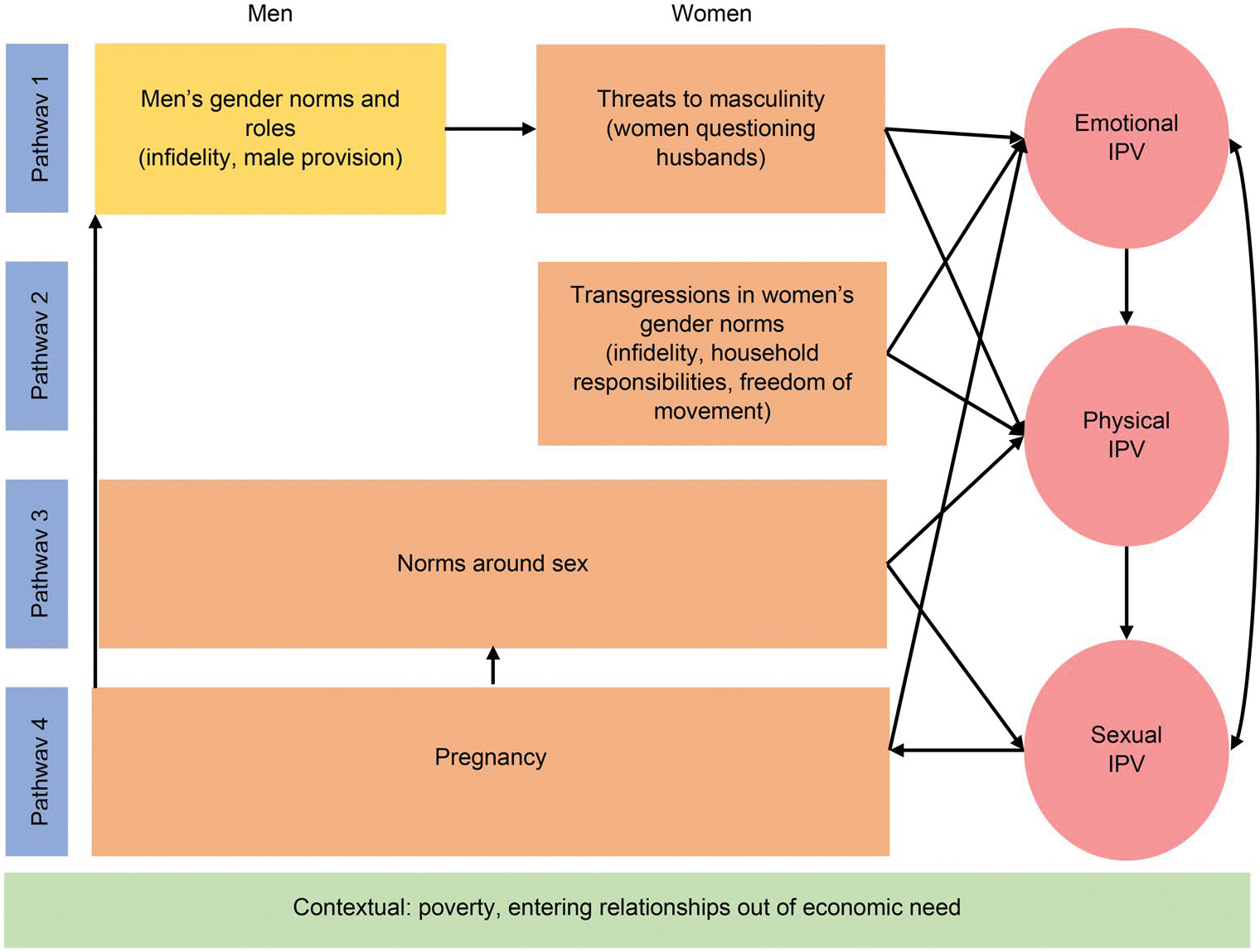
Pathways from stressors to IPV among young women in Malawi.

Figure 1 illustrates the pathways to IPV that we identified in the transcripts. Pathway 1 focused on emotional and physical IPV arising from threats to masculinity, such as women questioning their husband’s infidelity and lack of male provision. Pathway 2 captured how women’s transgressions of gender norms, including not fulfilling household responsibilities, real or suspected infidelity, or travelling without their husband’s permission, triggered emotional or physical IPV. Pathway 3 focused exclusively on norms about sex and sexual IPV. And Pathway 4 depicts how pregnancy led to Pathway 1 along with pregnancy arising from sexual IPV.

### Pathway 1: threats to masculinity → emotional or physical IPV

Women indicated that there were two stressors related to threats to norms around 1) men’s infidelity and 2) a lack of male provision.

Several women spoke about norms surrounding the unacceptability of having partners who ‘moved around’ or were seen with other women, who chatted with other women, or had girlfriends. Many women also shared that they did not trust their partners: ‘I feel he has other women apart from me, because of how he makes his moves’ (R4, 28 years), indicating that men sometimes came home late or not at all because they were spending time with other women. In addition to their own suspicions of husbands’ infidelity, women reported that rumours about their husbands’ affairs strained their relationships. One woman said, ‘Things that create tensions in our marriage is other people who come to tell me about my husband, that he has seen him with another woman and that they saw him giving her money, that creates tension’ (R33, 29 years), reflecting the dual stress of the affair and reduction in household resources. Another participant reported that her husband had slapped her ‘because [she] was asking too much about his cheating behaviour. Like why would someone act that way if it was not true? Then he ended up slapping me’ (R26, 26 years). This example highlights how husbands lashed out emotionally and physically when confronted about their affairs, which several women also reported.

Another woman recalled about how her husband’s infidelity put additional stress on her to provide for her family and explained that the norms on keeping silent in relationships was why she did not seek help:

So, he could leave in the morning and come back late hours, as a woman I have some money left what else could I have done? I could get the money and go to the market to buy some relish and cook for my children so when I meet him there he thought I am following him and that’s when he was getting angry…

Interviewer: Did you ever talk to someone about the issues you were facing?

Why should I? we don’t [talk] to anyone about family matters.… I haven’t ever told anyone of my situation except that time when we broke up. That’s when they knew the things I was passing through. (R35, 28 years)

Similar to the above respondent’s experience, a husband’s infidelity was a common reason why women left a relationship in our interviews.

Several women reported that their husband’s inability to provide food or soap for the household, strained their relationships. When asked about her relationship with her husband, one participant linked her relationship tensions to their household’s finances: ‘Most of the times it’s because of our poverty. When I ask him for basic needs such as soap and it should happen that he doesn’t have it, we end up quarrelling’ (R32, 27 years), revealing the pathway from lack of male provision and women’s consequent confrontation of their husbands’ behaviours that led to IPV.

Women’s frustrations about their husbands’ involvement with multiple women, whether affairs or polygamy, were also rooted in their husbands’ ability to provide for the household and the extra burden women faced in taking care of their families.

He has two wives, so sometimes when you tell him there is no food he doesn’t buy that’s where the problems originate and sometimes when you tell him I need money for fertiliser he says he cannot afford that’s where the problems originate. (R2, 26 years)

Men’s responsibilities to provide for multiple women meant that there was less money to go around, causing tensions in the household. Another woman stated, ‘he was not even providing food for us in the household, so I was just struggling with the children’ (R23, 25 years). Women reported that confrontations led to quarrels and experiences of emotional IPV, but none reported ensuing physical IPV. Similar to exiting relationships because of men’s infidelity, most participants noted that it was normatively acceptable for women to leave a relationship when their husbands did not provide for them, including when other women were involved:

If a man doesn’t take care of the woman. For example, not providing soap for the woman to wash her clothes, even if she possesses just three wrappers but she needs to wash them to be clean. Also, not providing for the household needs like relish and instead giving out excuses but later on found providing it for girlfriends. And when a wife finds out the marriage ends (R27, 19 years)

### Pathway 2: gender norms → emotional or physical IPV

Women reported that their failure to complete their household responsibilities, infidelity and related rumours, and mobility triggered IPV. For example, one woman reported that her husband had threatened to beat her because ‘[she] did not cook on time’ (R7, 21 years), while another shared: ‘I denied going to the farm saying I am tired and he said yesterday you did not go and today you don’t want let’s go, so I said I won’t go and he slapped me’ (R18, 23 years). These examples reveal how transgressing social expectations for women, such as cooking, cleaning, caring for children, and contributing to farming could trigger IPV experiences within this context.

However, one woman shared, ‘We tend to have arguments and disagreements [about money] … when this happens, I don’t cook for him and the children that day. So, my husband just cooks and eats with the children’ (R5, 26 years), reflecting how pathways 1 and 2 interact and how a lack of male provision affects women’s decisions to comply with gender norms.

Women also reported that their husbands wanted to know where they were and got angry when their husbands suspected or found out about their affairs:

He usually listens to the lies that his friends have told him about me, then when he has come back from the market at night, he just starts shouting at me without first asking if I did what he has been told… He has slapped me uncounted times… He insults me and slaps me when he has heard stories from his friends about me. He does not like to discuss when we have disagreements (R8, 22 years)

This example reveals how men did not first ask their wives about real or suspected affairs. Rather, men immediately insulted or slapped their wives, highlighting more severe consequences for transgressions of women’s gender norms compared to men’s transgressions.

Women’s conformity to gender norms helped mitigate men’s controlling behaviours. One woman explained how her husband did not prevent her from seeing family or friends because ‘when leaving the house I make sure my husband is aware’ (R34, 25 years). Women explained how broader social and gender norms contributed to IPV perpetration, ‘In this community when people see you [simply] standing with a man, they think it is a relationship’ (R18, 23 years), and that such misunderstandings around men’s and women’s interactions could be triggers for violence.

### Pathway 3: denying men sex → physical or sexual IPV

Sexual violence was commonly reported when women told their husbands they were tired or ‘not in the mood for sex’ (R32, 27 years). Women reported being upset after quarrels with their partners and not wanting to have sex, as one woman described ‘(in low tone) … after arguments. One day I was mad and did not feel like giving in but he forced himself on me against my will’ (R6, 23 years), illustrating the link between different forms of IPV, in this case emotional and sexual IPV.

Women also described how sex and sexual violence could be forms of control. When women denied their husbands sex, men became angry and sometimes resorted to violence: ‘When the man wants sex and I tell him no, I am tired, he will beat me’ (R18, 23 years). Two women reported that their partners forced them the first time they had sex, and one shared how she had been trying to avoid getting pregnant at that time.

### Pathway 4: pregnancy ←→ sexual IPV

Some women described how pregnancy brought about a change in their relationships: ‘We started disagreeing because he impregnated me, because the support changed, and he changed the way he was acting’ (R30, 19 years). Other women reported that their husbands had asked them to have an abortion, which they has refused, as described in the example below:

When I told him I am pregnant he said abort and I said I will not abort… He said I will not take care of your child… so I said I will do that no problem… just after being discharged from the hospital [he] stayed for one day … and he has not even brought soap or anything till now… At first everything was going well. Only after being pregnant did things change (R21, 24 years).

Other participants described experiences of IPV when they were pregnant. Shaming, humiliating, and insulting women about their pregnancies were commonly reported types of emotional IPV, which sometimes escalated into physical IPV:

He did shame me one time when I was pregnant…We were arguing, and he said something that he was going to marry another woman with no pregnancy, so I answered him back to carry on with his decision to go on - and he got mad that I’d answered him back. Then he gave me a slap (R6, 23 years).

The same participant ended up leaving her marriage, not because of her experience of IPV, but rather because her husband had asked her to have an abortion.

Pregnancy could also be an outcome of sexual IPV. One participant shared how her ex-partner, ‘forced me to sleep with him and he controlled me with his power and that is how I got pregnant of my child’ (R40, 22 years). She had not told anyone about this because she was afraid.

### Help-seeking and leaving relationships

Women disclosed IPV experiences to and sought help from family and friends, although such behaviours were more common for emotional or physical violence. One woman who had experienced physical violence after her husband believed rumours of her infidelity said that she talked to her sister, who ‘gave me advice on how I can approach him to end the violence’ (R8, 22 years). She further explained that she had not considered leaving her marriage because, ‘I have nowhere to stay now and have no one to help me financially’, reflecting her economic inability to leave her abusive marriage.

Cases of formal help-seeking were rare and not directly related to violence. For example, one woman sought help from the police when her husband failed to provide for her. Unlike the acceptability of leaving a relationship due to men’s infidelity or lack of male provision, women’s gender norm transgressions and experiences of violence were not viewed in the same light. When one young woman’s husband became angry with her for talking to another man, she sought help from an *ankhowse* [marriage counsellor] who told her ‘that what [she] did was wrong’ (R4, 28 years), further reflecting dominant relationship norms in this context.

Multiple women never disclosed violence because ‘that is between the both of us’ (R10, 23 years) or because ‘it is a secret’ (R21, 24 years), highlighting the norms surrounding the confidentiality about what takes place in marriage. Another woman explained that she had not sought healthcare as a result of violence ‘because I knew that it is nothing compared to what others were facing in their marriages’ (R39, 22 years) reflecting the normalisation of violence in this setting. Only one woman reported leaving her marriage due to IPV: ‘The beating, I said maybe one day he will beat me more than this… So I asked myself questions… Why am I suffering here as if my parents died. It is better I go home [to them]’ (R18, 23 years). A few women discussed how having children prevented them from leaving their marriages - ‘No, I have four children who will take care for them?’ (R15, 29 years) - reflecting the economic challenges with leaving abusive partners.

## Discussion

In this study, we identified four relationship pathways leading up to tensions and IPV, centred on threats to masculinity and women’s transgression of local gender norms. Our findings confirm the deeply ingrained nature of gender norms in the study setting, within a larger context of extreme poverty. Of note, despite all participants being raised in households that received cash transfers, the transfers were not a salient theme perhaps because young women did not receive the transfers themselves.

In our pathways related to norms, we found that young women’s economic vulnerability when entering relationships, and norms concerning male provision in relationships, highlight the complex nature of love and material exchange. In our study, young women purposely entered relationships to ensure their basic needs were met because they could not continue relying on their parents, similar to another study in this setting (Ansell et al. 2018). These experiences indicate lower bargaining power and higher instrumentality within relationships (Stoebenau et al. 2016). In the context of other cash transfer programmes examining the same age group, such as in South Africa, some young women also engaged in relationships for purely material motivations (Pettifor et al. 2016; Ranganathan et al. 2017). However, in our study and in South Africa, women also discussed the importance of love and men’s gender roles around provision in relationships, reflecting gift exchange, gender norms, and lower instrumentality in entering relationships (Stoebenau et al. 2016). Our findings reiterate that targeting exploitation in relationships to prevent IPV should consider the spectrum of deprivation, varying definitions of what constitutes transactional sex, and norms around this practise in each context (Stoebenau et al. 2016).

According to women’s accounts, pregnancy could be a source of tension and an IPV trigger. Previous research has identified mechanisms for increased IPV during pregnancy due to increased emotional and physical dependence, and relationship stress (Adjimi Nyemgah, Ranganathan, and Stöckl 2024). Few women in our study reported that health workers ever asked them about experiences of violence. Although respondents did not elaborate on this topic, insufficient resources for antenatal care or challenges in seeking care due to controlling or unsupportive partners are common barriers for IPV screening during antenatal care visits (Aboagye et al. 2022). While gender norms surrounding marriage and childbearing prevent women from leaving relationships in Malawi, women in our study also described the economic challenges of leaving abusive partners. Cash transfer programmes and other community- and facility-led interventions that can help identify women at risk of experiencing IPV and provide them with support to leave abusive relationships, as well as prevent IPV.

It was also clear from this study that specific triggers were related to particular types of violence. For example, poverty-related stress, women’s infidelity, or refusal to engage in household responsibilities often resulted in emotional and/or physical IPV. We found that triggers for sexual IPV were instead rooted in norms around sex. The distinct triggers for different forms of IPV indicate the need for tailored approaches within violence prevention programmes. While cash transfer programmes can alleviate poverty-related stress and ensuing emotional and/or physical conflict, targeting broader social norms, especially around women’s sexuality, are also needed to reduce and prevent sexual IPV. Evidence from studies of couples’ curricula shows promise in targeting gender norms related to sexual coercion in Rwanda (Stern and Heise 2019). In Tanzania, the Ujana Salama intervention within the national cash transfer programme improved gender-equitable attitudes among young men (Chzhen et al. 2021). In our study context of Malawi, the new Social Protection for Gender Empowerment and Resilience (SP-GEAR) programme aims to increase access to social services related to social behaviour change, sexual reproductive health and gender-based violence for girls and women in SCTP households (Malawi Government and UNICEF Malawi 2024). This combined programming addresses structural poverty while reaching girls with additional social services. In addition, social programming such as addressing harmful gender norms among both adolescent and young women and men through structured curricula or trainings, even before they enter relationships, will be crucial to prevent IPV.

### Limitations

Despite these rich findings, our analysis is subject to several limitations. First, we did not probe for detailed information about prior relationships as our interview guides focused on current or most recent relationships to minimise recall bias and respondent burden. We were limited to examining only what women themselves brought up, which limited our ability to develop a nuanced understanding about past relationships. Second, although steps were taken to ensure privacy during interviews and minimise respondent discomfort, IPV is a sensitive topic that is often underreported, especially due to social desirability bias. Although interviewers were trained extensively, respondent-interviewer power relations and interactions could have limited IPV reporting. Therefore, this study may not have captured experiences of those who have experienced IPV but chose not to disclose it. Third, those who are younger or more economically dependent on households may have a different vulnerability to IPV which we cannot capture, although we did not find differences by age group in these interviews. And finally, our findings are specific to the study setting, and we caution against generalising them to other contexts. However, the rich, in-depth nature of the data does facilitate comparison and hints at transferable insights that could be explored in other settings.

## Conclusions

This study adds to growing evidence on how economic vulnerability interacts with gender and social norms early in relationships in the context of the Malawi SCTP. The lack of intergenerational effects of cash transfers on young women’s relationships suggests that cash alone may not be sufficient to effectively reach the next generation. We also found that while poverty-related stress often triggered emotional and/or physical IPV, sexual IPV experiences were deeply rooted in local gender norms. Therefore, we posit that cash transfer programmes can be layered with targeted interventions, such as training and curricula for youth and adolescents on harmful gender norms, thus addressing group-specific vulnerabilities. Future research should first effectively measure gender attitudes and norms among adolescents and then identify which interventions would be an appropriate response to context-specific findings. Overall, our findings indicate the need for future programmes to tackle poverty and harmful gender norms together to prevent IPV early in relationships. Examining the precise combination of cash and norms programming among this demographic also requires further study.

## Supplementary Material

Supp 1

Supplemental data for this article can be accessed online at https://doi.org/10.1080/13691058.2025.2609888.

## Figures and Tables

**Table 1.** Stratification of participants by treatment status, age group, and district.

	Control (late cash transfer receipt)		Treatment (early cash transfer receipt)		Total

District	19–24 years	25–29 years	19–24 years	25–29 years	
Salima	6	5	5	5	21
Mangochi	5	5	5	5	20
**Total**	11	10	10	10	**41**

Notes: From a total of 41 interviews, two interviews were not analysed because of translation issues. Both interviews used interpreters from the community and interviewers noted potential response bias in their field notes. Treatment households in the Malawi Social Cash Transfer Programme received the cash transfer in 2013. The control group was rolled into the programme in 2016

**Table 2.** Descriptive characteristics of the sample (*n* = 39).

Indicator	Mean (range or N)

Age (years)	24.5 (range: 19–29)
Some secondary education	23.1% (*N* = 9)
Partner's age* (years)	30.3 (range: 19–42)
Current union	69.2% (*N* = 27)
Have living children	79.5% (*N* = 31)
Number of children (among those who had children)	1.9 children (range: 1–4 children)
*Experience of*	
Emotional IPV	26.3% (*N* = 10)
Physical IPV	18.4% (*N* = 7)
Sexual IPV	34.2% (*N* = 13)
Physical and/or sexual IPV	42.1% (*N* = 16)

Notes: *If known. *N* = 10 unknown. IPV = Intimate Partner Violence. One woman who had recently been widowed was not asked the IPV questions

## Data Availability

The data from this study are not publicly available as they contain sensitive participant information.
