# Supplementary material for: Exploring relationship pathways to prevent intimate partner violence among young women in Malawi
Source: Cult Health Sex. Author manuscript; Available in PMC 2026 Mar 30. (PMC13033620; doi:10.1080/13691058.2025.2609888)
Supplement: Supp 1 [file NIHMS2134574-supplement-Supp_1.docx]

# Appendix A: Relationship pathways

Note: The examples below depict the relationship pathways described in the interviews. They were developed to better understand relationships and guide the development of our codebook. We created one pathway map for each respondent, considering the context of entering relationships, relationship stressors or triggers, IPV experiences, help-seeking behaviours, and reasons for exiting relationships. Arrows indicate how respondents themselves linked events, while dotted lines indicate other events described but without that link.

For example, in Example 1, the respondent discussed entering her current relationship out of economic need and with her family’s approval. The use of money within her relationship was a trigger for emotional IPV, but she did not seek help as she said there was no one to help her. In Example 2, the respondent did not enter the relationship out of economic need. She explained that transgressing gender norms by being disrespectful to her husband had stressed the relationship. Violence then began when she became pregnant, but she did not seek help at that time because she did not know where to go. She left the marriage when her husband stopped providing for her, at which time she sought help from his family, and she and her husband agreed to end their marriage.

## Example 1

current

relationship

use of

money

emotional

IPV

family approved relationship

no help seeking

(no one to help)

entered relationship

out of economic need

triggers of IPV

relationship context

IPV

help-seeking behaviours

relationship exit

**Key:**

***Example 2***

former

relationship

woman

disrespecting

husband

pregnancy

non-IPV

help

seeking

husband’s

affairs

emotional

IPV

physical

IPV

lack of male

provision

exit

no help-seeking

(didn’t know where to go at that time)

sexual

IPV

did not enter relationship

out of economic need

***Example 3***

lack of male

provision

controlling

behaviours

informal & formal help-seeking

current

rumours of woman’s infidelity

men’s sexual performance

emotional IPV

physical IPV

sexual IPV

considered leaving marriage

left to

mother’s house

still in marriage with conditions

other

entered relationship

out of economic need

family approved

relationship
